# Investigating the feasibility and acceptability of the TeleRehabilitation of balance clinical and economic Decision Support System (TeleRehaB DSS) in adults at risk of falls: study protocol for a multicentre clinical trial

**DOI:** 10.1136/bmjopen-2025-108821

**Published:** 2026-06-25

**Authors:** Brooke Nairn, Isabelle D Walz, Christos Nikitas, Diego Kaski, Nattawan Utoomprurkporn, Christoph Maurer, Dimitris Kikidis, Vassilis Tsakanikas, Dimitrios Fotiadis, Marousa Pavlou, Doris-Eva Bamiou

**Affiliations:** 1 The Ear Institute, University College London, London, UK; 2 The Institute of Neurology, Department of Clinical and Movement Neurosciences, University College London, London, UK; 3 Department of Neuro-Otology, Royal National Ear Nose Throat Hospital, University College London Hospitals, London, UK; 4 Department of Neurology and Neuroscience, University of Freiburg, Faculty of Medicine, Freiburg, Germany; 5 Department of Sports and Sport Science, University of Freiburg, Freiburg, Germany; 6 First Department of Otorhinolaryngology, Head and Neck Surgery, National and Kapodistrian University of Athens, Athens, Greece; 7 Faculty of Medicine, Chulalongkorn University, Bangkok, Thailand; 8 Chulalongkorn University Faculty of Medicine, Bangkok, Thailand; 9 Hearing Health Theme, NIHR University College London Hospitals Biomedical Research Centre, London, UK

**Keywords:** Artificial Intelligence, Augmented Reality, Telemedicine, Brain Injuries, Clinical Trial, Neurology

## Abstract

**Introduction:**

Falls are a significant concern for older adults, particularly those with neurological, vestibular, cognitive and post-viral conditions, due to dizziness and imbalance. Conventional balance rehabilitation programmes, though effective, face challenges in adherence and accessibility. The TeleRehabilitation Decision Support System (TeleRehaB DSS) uses artificial intelligence (AI) and motion tracking to provide individualised multisensory balance rehabilitation (MBR) remotely. This trial aims to evaluate the acceptability, feasibility, safety and preliminary efficacy of a home-based TeleRehaB DSS among community-dwelling older adults at risk of falls due to stroke, mild cognitive impairment (MCI), long COVID and vestibular dysfunction.

**Methods and analysis:**

This multicentre, assessor-blinded randomised controlled trial will recruit 460 community-dwelling adults aged 40–80 years with stroke, MCI, vestibular dysfunction or long covid across five sites in the UK, Europe and Southeast Asia. Participants will be randomised to a 9-week remotely supervised home exercise programme using either TeleRehaB DSS (high-tech or low-tech MBR with exergames and cognitive training) or standard care (OTAGO home exercise programme or Meniere’s Dizziness booklet). Primary outcomes include feasibility, acceptability and safety, alongside clinical measures of balance and health-related quality of life (Functional Gait Assessment, EuroQol five-dimensional descriptive system instrument). Secondary outcomes assess balance, cognition, physical activity, dizziness, psychological well-being, fatigue and confidence. Usability, user experience and digital health literacy will also be evaluated. Anonymised data will undergo quality checks and be analysed using descriptive and exploratory statistics, mixed-effects models, cost-effectiveness analysis (incremental cost-effectiveness ratio) and thematic analysis of qualitative interviews, adjusting for site and relevant confounders.

**Ethics and dissemination:**

This study has received institutional ethical approvals in the UK, Germany, Greece and Thailand and from Madeira, Portugal. Findings from this study will be submitted for peer-reviewed publications.

**Trial registration number:**

NCT06534164.

**STRENGTHS AND LIMITATIONS OF THIS STUDY**

- This multicentre, assessor-blinded randomised controlled design strengthens internal validity while assessing the feasibility and acceptability of the TeleRehabilitation Decision Support System (TeleRehaB DSS) intervention compared with a standard home exercise programme.
- The mixed-methods approach enables triangulation of quantitative usage metrics with qualitative user experiences to comprehensively assess acceptability and usability.
- The multicentre recruitment across several international sites may introduce variability in implementation and participant support despite use of a standardised protocol.
- As a feasibility study, the sample size is not powered to detect clinical effectiveness outcomes.
- Reliance on participant self-setup and home use of technology may influence adherence and data completeness.

## Introduction

### Falls epidemic

 Falls are a rising epidemic among older adults, occurring annually in 30% of adults aged over 65 years, with wide-ranging physical, psychosocial and healthcare-related consequences.^1 2^ The Global Burden of Diseases study reported nearly 17 million years of life lost from falls in 2017, with the expected number of falls and related injuries likely to further increase with the ageing population.^3^,^5^

Falls are multifactorial, especially during performance of complex tasks, when falls are most likely to occur.^6^ Impaired balance and dizziness have consistently been identified as risk factors for falls which are associated with multiple comorbidities, including stroke, mild cognitive impairment (MCI), vestibular dysfunction and long COVID.^7^,^10^ Balance and mobility problems are among the most frequent and disabling effects of stroke, with two-thirds of survivors experiencing residual degrees of disability^11^,^13^ with falls as one of the most commonly resulting consequences.^14 15^ Although less studied in middle-aged adults compared with older adults, falls are a significant issue among both groups,^16^ with studies indicating that fall rates remain high in middle-aged adults post-stroke, particularly among those with residual functional limitations.

Compromised cognitive function, as in individuals with MCI and/or long COVID, is also associated with increased risk of falls due to reduced executive function and attention and slow processing speeds which affect balance and gait.^7 17 18^ Adults with MCI have a notably higher incidence of falls compared with those with normal cognitive function.^7 18^ Similarly, emerging evidence has found that the long-term effects of COVID-19, known as long COVID, can include persistent symptoms of cognitive decline, fatigue, muscle weakness and balance issues, all contributing to an increased risk of falls.^10 19 20^ Furthermore, vestibular disorders, including central (stroke, multiple sclerosis, cerebellar disease) and peripheral (i.e., unilateral vestibular hypofunction, vestibular neuritis, labyrinthitis) causes, directly impair balance and spatial orientation, significantly increasing the likelihood of falls.^9 21 22^

### Balance rehabilitation

Balance is complex, relying on integration of vestibular, visual and somatosensory inputs for postural orientation and motion while central processes monitor and control the interaction between musculoskeletal and neural systems to generate anticipatory postural adjustments and adapt posture to changing environmental and balance task demands.^1423^,^25^ Previous studies have indicated that multisensory balance rehabilitation (MBR) can improve dynamic balance.^23^,^27^ MBR consists of exercises that aim to improve balance, gait, somatosensory integration and gaze stability through compensation, adaptation, habituation and/or substitution of the vestibular ocular reflex and/or vestibulospinal reflex.^28^,^30^ Multisensory exercises, consisting of activities simultaneously stimulating at least two sensory modalities, including visual, auditory, tactile, vestibular and somatosensory systems, are effective for improving balance in people with balance disorders.^24^ MBR programmes which address balance have shown substantial reduction in falls risk in older adults, compared with standard programmes.^231^,^33^

Balance rehabilitation is the most effective treatment for balance disorders across all ages, typically involving personalised exercises prescribed by healthcare professionals.^21 34 35^ However, access to specialist services is often limited due to resource and staffing constraints. While effective in reducing fall risks among older adults, balance/falls programmes face challenges such as poor adherence, with up to 50% of participants dropping out early.^3436^,^38^ Common limitations include inadequate exercise progression, lack of individualisation and minimal supervision, and they often fail to address real-world situations such as dual-task training or activities simulating everyday challenges (eg, crossing a road).^1734 36^,^38^

Studies concerning the effect of MBR on balance and gait outcomes among stroke survivors, MCI, vestibular dysfunction and long covid are limited,^27^ and even in persons with vestibular dysfunction where multiple studies exist, optimal treatment strategies are still unknown.^9 21 39 40^ Balance rehabilitation programmes are rarely multisensory and commonly lacking a cognitive element, which are key factors in addressing static and dynamic balance.^17 41 42^ Further research is needed to investigate the optimal MBR for these populations.^43^

### Digital health technologies

Recent advancements in digital health technologies have enabled innovative approaches to rehabilitation delivery, including remote and home-based interventions.^44^ Telerehabilitation, the remote provision of rehabilitation by means of information and communication technologies (ICT), provides a promising solution to address these challenges. Emerging evidence suggests that ICT, such as wearable sensors, computerised decision support systems, extended reality (augmented or virtual reality) and telerehabilitation platforms, have the potential to improve clinical efficiency and satisfaction and enhance care quality, while reducing healthcare costs.^45^ Compared with traditional in-person training, telerehabilitation has been shown to improve patient accessibility while overcoming socioeconomic, geographical and cultural challenges.^43 46 47^ Furthermore, data from the HOLOBalance project have shown improved balance and gait outcomes in non-neurological fallers, following remotely delivered MBR via augmented reality (AR).^47^

The TeleRehabilitation Decision Support System (TeleRehaB DSS), evolved from its predecessor, HOLOBalance,^48^ leverages artificial intelligence (AI) and motion tracking with real-time feedback to offer remotely delivered individualised MBR via AR, addressing the lack of expert balance physiotherapists. Delivered in the comfort of patient’s home, it offers an interactive rehabilitation programme incorporating (1) functional training, (2) multisensory balance exercises, (3) cognitive training and (4) gamified activities (exergames).

### Aims

TeleRehaB DSS will use off-the-shelf technologies to enable remotely monitored MBR for adults at risk of falls due to either stroke, MCI, vestibular dysfunction or long covid. This study protocol outlines the global, multisite randomised controlled trial (RCT) that aims to (1) determine the system’s safety, acceptability and feasibility; (2) assess how quality of life (QOL), balance function and confidence, cognitive function and well-being compare to standard of care interventions following a 9-week balance programme; and (3) provide preliminary data for a definitive randomised controlled trial.

## Methods

### Trial design

**Figure 1:**
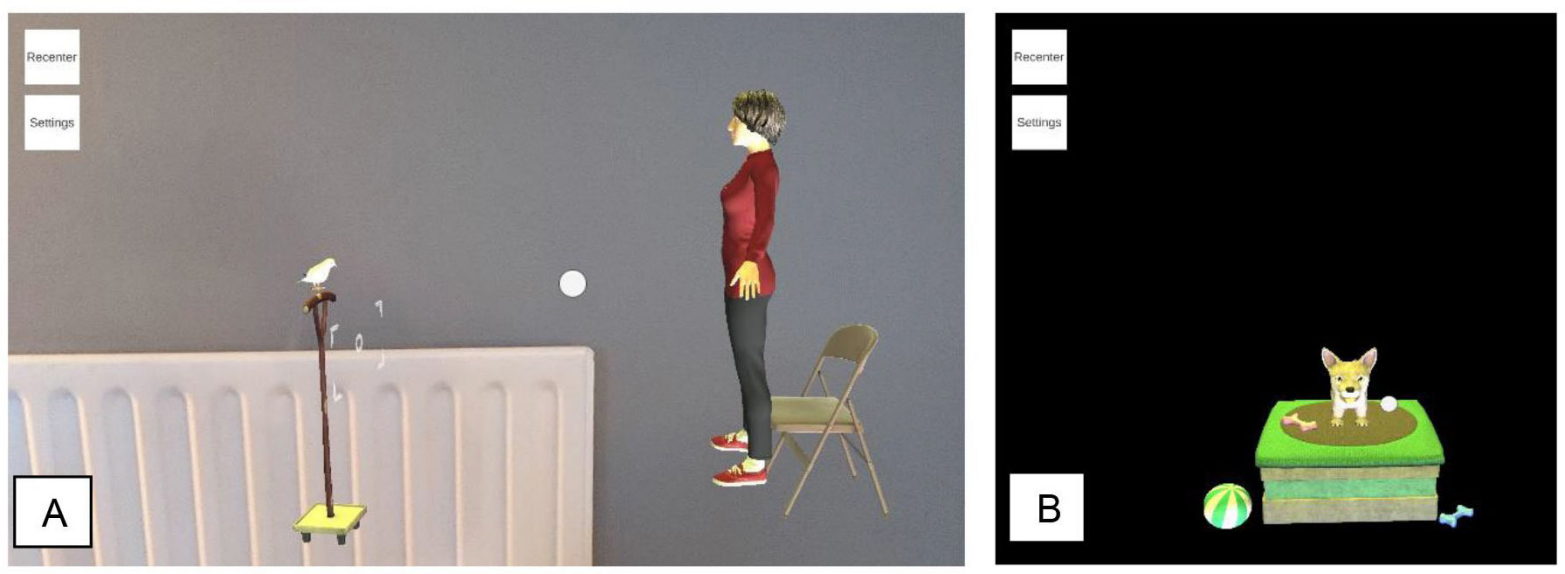
Screenshots of (**A**) physiotherapist avatar and (**B**) representative exergame from the TeleRehab DSS. TeleRehab DSS, TeleRehabilitation Decision Support System.

The study is a multicentre, assessor-blinded RCT, investigating the feasibility and acceptability of the TeleRehaB DSS in persons with stroke, MCI, vestibular dysfunction or long covid.^49^ All participants will be enrolled in a 9-week MBR home exercise programme (HEP), including individualised balance exercises (sitting, standing, walking), cognitive training and exergames (figure 1). All participants will be randomised into one of three study arms, where they will receive a HEP provided under the supervision of a clinician (physiotherapist or sports scientist): (1) the high-technology TeleRehaB DSS group (HT-IG), (2) the low-technology TeleRehaB DSS group (LT-IG) or (3) the control group (CG) standard of care HEP. No matter the study arm allocated, all participants will receive the same prescription pattern, receiving a weekly check-in call by a member of the research team, and remote programme reviews at weeks 3 and 6 by the treating clinician, to discuss exercise modifications, symptoms, falls and any technical problems.

Exercise data will be collected automatically in TeleRehaB DSS dashboard for the HT-IG and LT-IG, whereas CG participants will be required to log their exercise data manually in a diary provided. Exercises will be prescribed and progressed according to participant feedback and task performance, as assessed by the treating clinician. All treating clinicians will receive appropriate training prior to trial onset.

**Figure 2:**
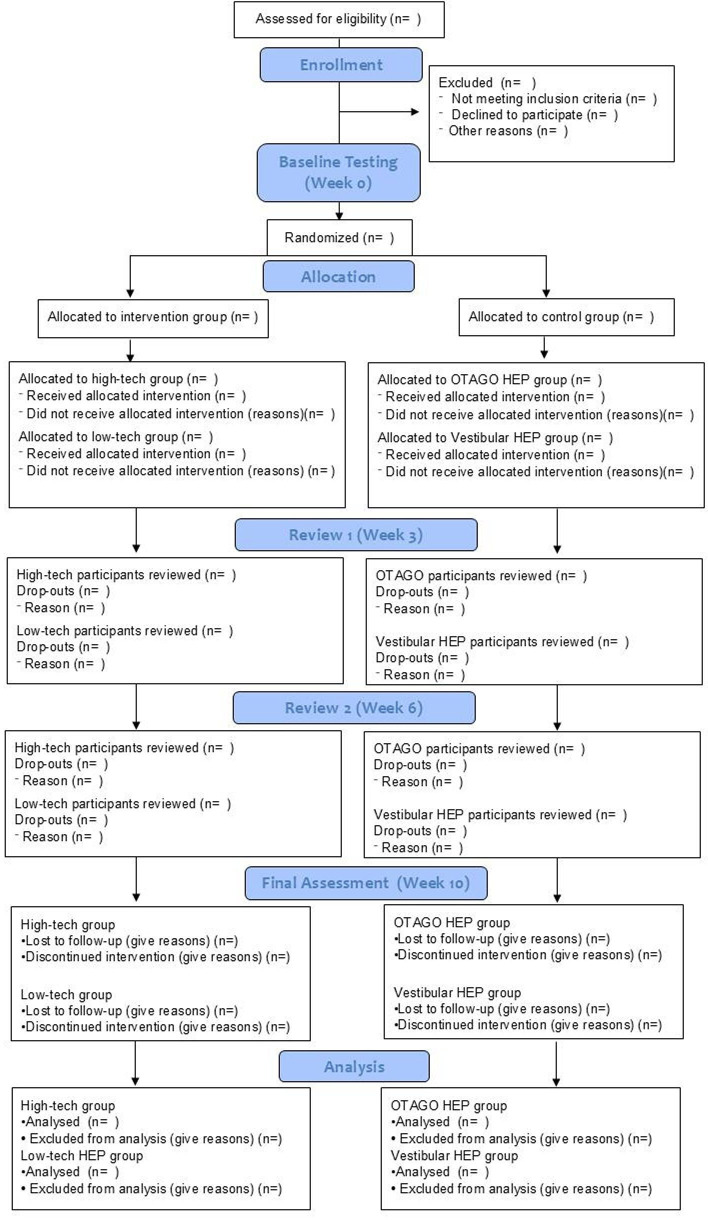
CONSORT diagram to demonstrate participant flow through the TeleRehaB DSS RCT. After baseline testing and randomisation, participants were allocated to either the intervention group, comprising a high-technology arm and low-technology arm, or the control group. The control group consisted of participants receiving either the OTAGO HEP or a vestibular-specific HEP. Assessments were conducted at baseline, week 3 (review 1), week 6 (review 2) and week 10 (final assessment). Participant numbers, dropouts and reasons for loss to follow-up or intervention discontinuation were recorded at each stage. Final analysis was performed according to group allocation, noting any exclusions. CONSORT, Consolidated Standards of Reporting Trials; HEP, home exercise programme; RCT, randomised controlled trial; TeleRehaB DSS, TeleRehabilitation Decision Support System.

This study will compare acceptability of the TeleRehaB DSS programme (adherence, recruitment and dropouts) compared with current standard of care (OTAGO or Balance Retraining Booklet), which are commonly used in balance and vestibular rehabilitation. This study will also explore trends for effectiveness across several validated clinical outcome measures to identify whether a future trial is warranted and if so, to provide sample size estimates. The protocol has been developed using the Standard Protocol Items: Recommendations for Interventional Trials reporting guidelines^50^ and the flow of participants through the trial will be recorded in compliance with the Consolidated Standards of Reporting Trials statement^51^ (figure 2).

### Study setting

The study will take place across five clinical sites (London, UK; Freiburg, Germany; Athens, Greece; Madeira, Portugal; and Bangkok, Thailand).

### Participants

Independently living, community-dwelling older adults aged 40–80 years who are at risk of falls, and clinically diagnosed with either stroke, MCI, vestibular dysfunction or long covid, will be recruited across the five clinical sites.

#### Inclusion criteria

All participants must meet the below criteria for consideration in the study, with additional specific criteria for each disease cohort (table 1).

**Table 1:** TeleRehaB DSS clinical study inclusion criteria

All participants	Age 40–80 years. Community dwelling able to walk 500 m independently or with a stick. HADS depression subscale score <10/21. No significant visual impairment (as self-reported by participants). Willing to comply with study procedures, proposed training and testing regime. Able to understand and consent to the research. Has at least one functional hand for grip function and computer use. Fulfilling all of the criteria from one of the below subgroups
Stroke participants	Individuals with diagnosis of focal ischaemic or haemorrhagic stroke, as confirmed by a clinical letter. Onset >6 months prior to study. MoCA score >23. FGA score <22/30 AND/OR having experienced a fall(s) in the last 12 months. If diagnosed with drop foot, must have a corrective device (AFO, FES etc.) must be in-place for exercise performance.
MCI participants	Individuals with diagnosis of MCI, according to the ICD10 (G31.84),^92^ as confirmed by a clinical letter. FGA <22/30 AND/OR having experienced a fall(s) in the last 12 months.
Vestibular dysfunction participants	Individuals with a diagnosis of a vestibular disorder (peripheral and/or mixed peripheral and central) as confirmed by a clinical letter: Peripheral vestibular disorder in which the balance problem lies in the vestibular/balance system within the inner ear. Mixed vestibular disorder in which the balance problem lies in the vestibular/balance system within the inner ear (peripheral) and involving the nerves or neuronal network in the brain/brainstem responsible for balance (central). Chronic dizziness and/or unsteadiness (>3 months duration) due to a vestibular disorder. DHI score >34 AND/OR FGA score <22/30. MoCA score >23.
Long covid participants	Individuals with laboratory confirmed diagnosis of COVID-19 (>6 months prior to study onset) AND/OR who have been diagnosed with long covid, as confirmed by a clinical letter. Who have chronic dizziness and/or unsteadiness which started after the COVID-19 illness (self-report by the patient; duration >3 months). DHI score >34 AND/OR FGA score <22/30. MoCA score >23.

AFO, ankle-foot orthoses; DHI, Dizziness Handicap Inventory; FES, Functional Electrical Stimulation; FGA, Functional Gait Assessment; HADS, Hospital Anxiety and Depression Scale; ICD10, International Classification of Disease 10; MCI, mild cognitive impairment; MoCA, Montreal Cognitive Assessment; TeleRehaB DSS, TeleRehabilitation Decision Support System.

#### Exclusion criteria

Participants will be excluded from the study if they present with the below exclusion criteria:

- HADS depression subscale score >10.
- Significant visual impairment, homonymous hemianopia or visual-spatial neglect (stroke cohort only) as self-reported.
- Uncontrolled hypertension.
- Diagnosis of other neurological problems (e.g., Parkinson’s disease, multiple sclerosis, etc.).
- Language and communication deficits impairing the ability to express thoughts (e.g., aphasia).
- Has participated in a clinical drug trial in the past 6 months.
- Has an acute musculoskeletal injury that prevents participation in a structured exercise programme (eg, lower limb fracture).
- Is currently or has within the past 8 weeks received falls/balance/vestibular and/or cognitive rehabilitation.
- Has an implanted electrical medical device or cardiac pacemaker.
- Diagnosis of unstable Meniere’s or more than four migraines/month at the time of participating in the study (vestibular cohort only).

#### Sample size

The target sample size across all sites is 460. This equates to 60–100 participants per clinical site, with 30–50 participants recruited to intervention group (IG) and CG study arms, per clinical site, in line with recommendations for pilot feasibility studies.^49 52 53^

#### Recruitment

Each clinical site will recruit participants via (1) web-based advertisements, (2) social network groups, (3) relevant local patient bodies (for stroke, MCI, balance/dizziness problems, long covid), (4) specialist consultants and/or (5) research staff. Potential participants will undergo a telephone screening session with a researcher to determine their eligibility, with eligible participants booked in for baseline testing with the blinded outcome assessor. Participants will provide written informed consent (online supplemental appendix 1) upon arrival at their baseline outcome assessment session.

#### Randomisation and blinding

**Figure 3:**
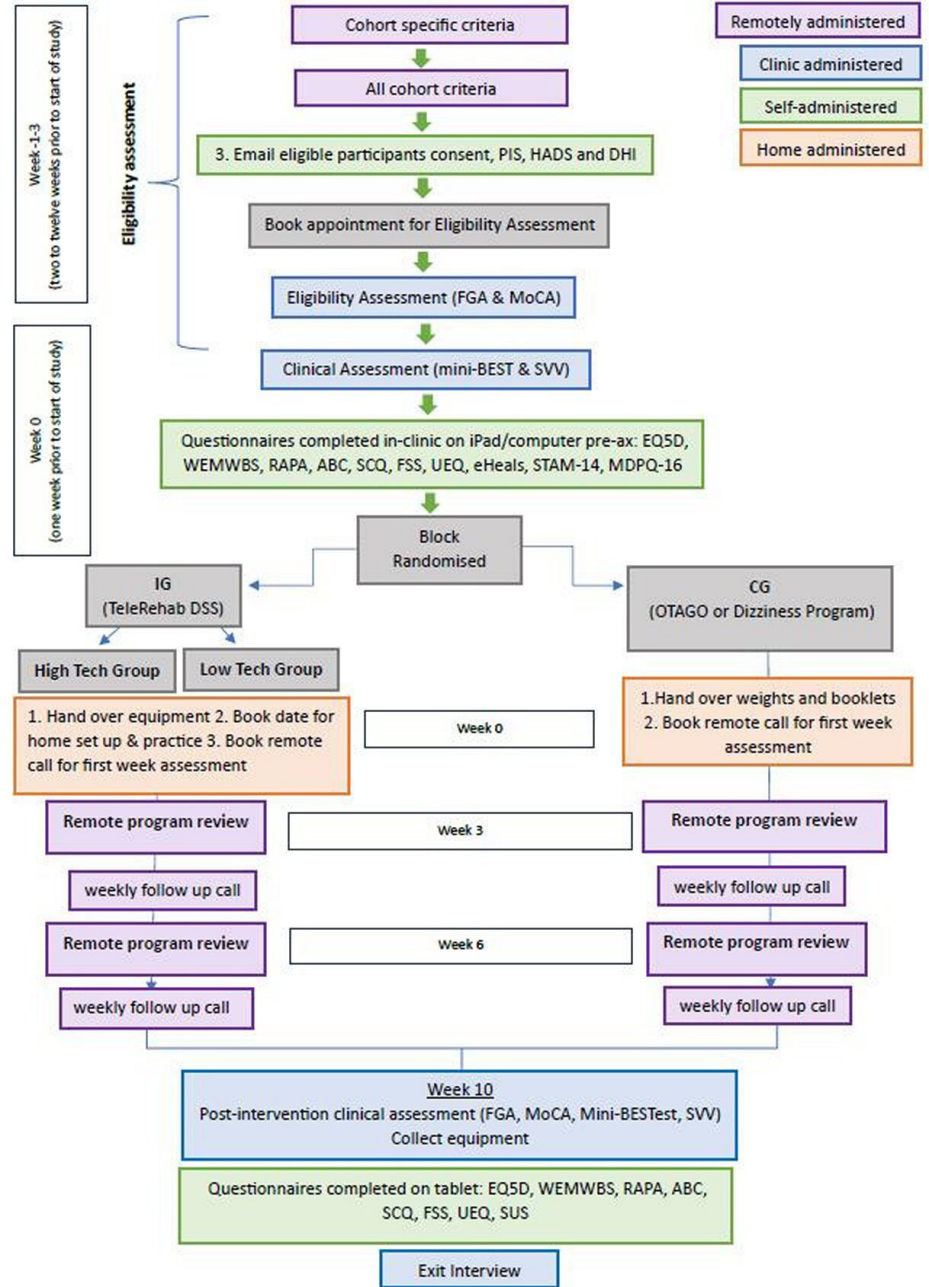
Flowchart of randomisation procedure and clinical flow of participants through the TeleRehaB DSS RCT. ax, assessment; ABC, Activities-Specific Balance Confidence Scale; CG, control group; DHI, Dizziness Handicap Inventory; eHeals, eHealth Literacy Assessment; EQ5D, EuroQuol-5 dimensions; FES, Functional Electrical Stimulation; FGA, Functional Gait Assessment; FSS, Fatigue Severity Scale; HADS, Hospital Anxiety and Depression Scale; IG, intervention group; MDPQ-16, Mobile Device Proficiency Questionnaire-16; Mini-BESTest, Mini-Balance Evaluation Systems Test; MoCA, Montreal Cognitive Assessment; PIS, Participant Information Sheet; RAPA, Rapid Assessment of Physical Activity; SCQ, Situational Characteristic Questionnaire; STAM-14, Senior Technology Acceptance and Adoption Model-14; SUS, System Usability Scale; SVV, Subjective Visual Verticality; TeleRehaB DSS, TeleRehabilitation Decision Support System; UEQ, User Experience Questionnaire; WEMWBS, The Warwick-Edinburgh Mental Wellbeing Scale.

Participants’ will be randomised using an online platform (www.sealedenvelope.com), and opaque envelopes, into either the HT-IG, LT-IG or CG to undergo a HEP. Participants will not be able to choose their preferred group and must be willing to participate in the assigned group. Details of the randomisation procedure, eligibility screening and intervention procedure can be found in figure 3.

This is a single-assessor blinded RCT. As this study is comparing a technology intervention (TeleRehaB DSS) versus standard care, it is not possible to blind the participants or the treating clinicians to the intervention provided.

### Intervention

#### Intervention group: TeleRehabilitation Decision Support System (high-tech and low-tech intervention arms)

Participants randomised into the TeleRehaB DSS IG will undergo a second randomisation into either the (1) high-tech group or (2) low-tech group.

**Figure 4:**
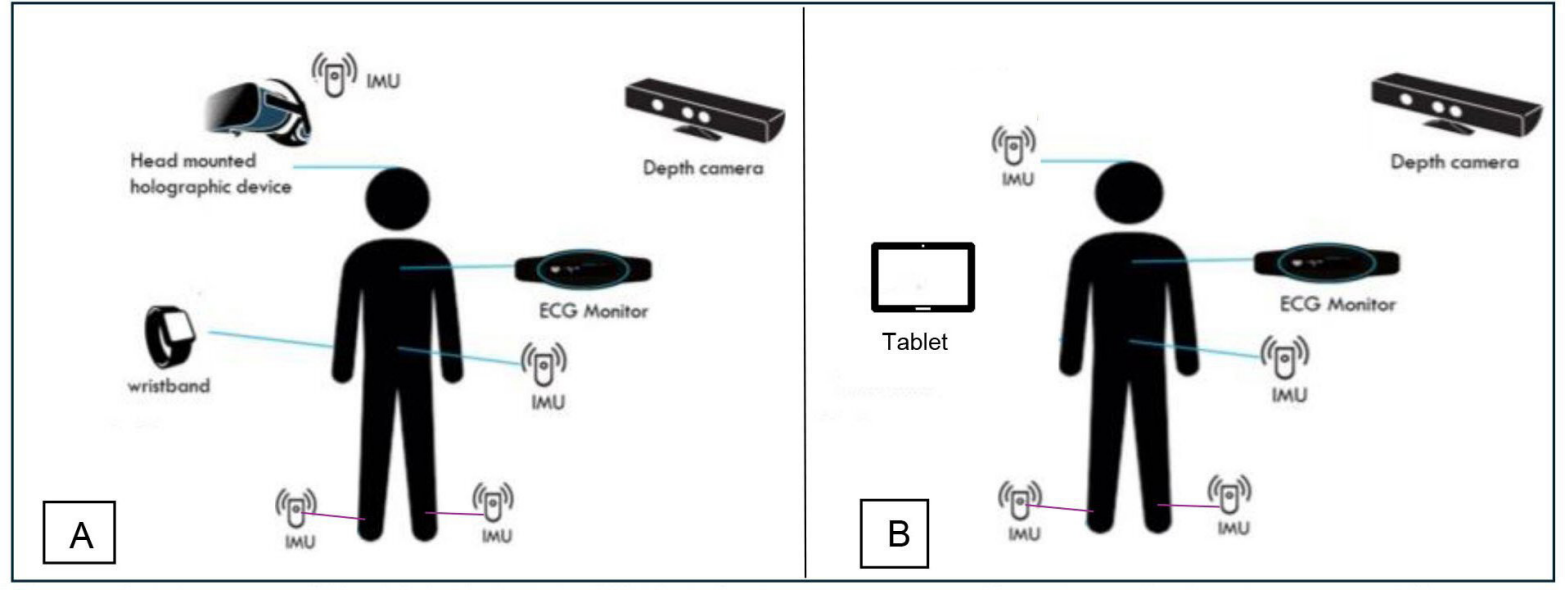
Diagram of the TeleRehabilitation Decision Support System (TeleRehaB DSS) high-technology arm (**A**) and low-technology arm (**B**) hardware. In the high-technology arm (**A**), participants use a head-mounted augmented reality (AR) device; four inertial measurement units (IMUs) placed on both ankles, head and trunk; a depth-sensing camera for full-body motion capture; a smartwatch for additional physiological data and an ECG monitor worn on the chest. In the low-technology arm (**B**), participants use a tablet for exercise display, the same four IMUs and ECG monitor, and the depth camera for motion capture.

The HT-IG will receive all components of the TeleRehaB DSS, consisting of a depth camera, lightweight AR headset that displays the hologram, inertial measurement unit (IMU) body-worn sensors, to record movement, a chest heart rate monitor and smartwatch for activity monitoring (figure 4). The AI-DSS will suggest multisensory balance exercises, including cognitive training and exergames delivered by AR. The platform also provides real-time feedback on individual’s performance based on collected IMU data, which are analysed using an EDGE computer processing unit. The LT-IG will receive a basic version of TeleRehaB DSS, with a depth camera, tablet and IMU sensor monitored exercises with real-time feedback, multisensory balance exercises and cognitive games, but without the exergames, smartwatch, mobile phone or AR.

The TeleRehaB DSS includes an extended version of the HOLOBalance rehabilitation programme delivered via an AR hologram of a virtual physiotherapist. It is based upon established, evidence-based MBR protocols that have been shown to improve balance control in older adults at risk of falls and in persons with vestibular disorders.^23 54 55^ Additional exercises have been added to the TeleRehaB DSS, including range of motion stretching exercises and optokinetic stimulation exercises for participants with visual induced dizziness. Please see online supplemental material table S2 for exercise details and progression levels.

IG participants will be visited by a research team member to instal the TeleRehaB DSS into their home and taught how to use the system, with a practice session. The initial set of exercises will be set by the treating clinician and all subsequent exercise progressions will be suggested by the AI-DSS and either approved or disapproved by the treating clinician. The approved exercise programme will be loaded into the participant’s training plan which will automatically be deployed.

Participants will be required to complete the programme 5 days per week over 9 weeks. They will be encouraged to complete the programme in one session per day, but if required due to fatigue and/or scheduling issues, may complete the session in multiple bouts within a 24-hour period. Participants will be required to wear the equipment when performing the prescribed exercises. At the end of the study, a subset of participants will be invited to participate in a semistructured exit interview to discuss their experience using the TeleRehaB DSS system. This qualitative feedback on acceptability and usability will help to inform future iterations of the platform, for a larger-scale RCT and eventual adoption into local healthcare systems.

#### Control intervention: OTAGO home exercise programme; Meniere’s Dizziness programme

CG participants will receive a standard of care balance rehabilitation HEP; stroke and MCI participants will complete the *OTAGO HEP*^56^,^58^ (accessible at https://www.livestronger.org.nz/assets/Uploads/acc1162-otago-exercise-manual.pdf), while vestibular and long covid participants will complete the *Balance Retraining Booklet*^59 60^ (accessible at https://www.meandve.org.uk/files/pdfs/Balancing_Retraining_(Nov_2023).pdf).

Participants will be required to log their exercises daily on a written diary. The exercises will be reviewed remotely by the treating clinician every 3 weeks. Participants will also be required to complete a falls diary (online supplemental material table S2), which will be reviewed in the weekly telephone call. Exercises will be progressed in line with the recommendations from the OTAGO HEP and/or Meniere’s Dizziness booklet, in combination with the treating clinician’s clinical judgement.

### Outcome measures

All study participants will attend two sessions at the clinical site: at baseline (week 0) and post-intervention (week 10), where a blinded outcome assessor will collect outcome measure data. Participants will be asked to complete a subjective questionnaire set, walking/balance tests, a verticality perception test and a cognitive screen. These will take approximately 1.5 hours, including comfort breaks. Participants’ e-health literacy will only be collected at baseline, while system usability score (SUS) and User Experience Questionnaire (UEQ) will be collected post-intervention among the IG only.

The outcome assessor will be asked to record any incidences of unblinding and detail how this occurred. Due to the nature of this study, we do not foresee circumstances where emergency unblinding of the outcome assessor is required.

#### Primary outcome measures

Primary outcomes of feasibility, acceptability, usability and safety will be investigated alongside clinical measures of Functional Gait Assessment (FGA) and EuroQol five-dimensional descriptive system (EQ-5D-5L) instrument pre-intervention and post-intervention. The FGA includes 10 tasks that assess dynamic and complex gait (eg, walking with head turns or stopping and turning), with a minimal detectable change (MDC) of 4.2 points in stroke populations and 6 points in vestibular disorders^61^ and minimally clinically important difference (MCID) of 4 points in community-dwelling older adults.^62 63^

Programme effectiveness will be evaluated using quality-adjusted life years (QALYs) measured with the EQ-5D-5L instrument.^64^ The EQ-5D-5L is a standardised, valid and reliable measure of health status for clinical and economic appraisal.^64^ The respondent is asked to rate their health status on five dimensions (mobility, self-care, usual activities, pain/discomfort, anxiety/depression), followed by a self-rated health score from 0 to 100. MCID ranges from 0.03 to 0.52.^65^,^67^

Primary outcomes are as follows:

1. *Acceptability*: recruitment (percentage of eligible participants enrolled); dropout rates; intervention adherence (percentage of prescribed sessions completed) monitored automatically through the system for TeleRehaB DSS participants and via an exercise diary for control groups.
2. *Safety*: participants will be monitored for adverse event’s via completion of adverse events forms on the system database, during telephone or face-to-face contacts as part of the intervention phase and during follow-up assessments. Falls diaries will be collected weekly from participants during the intervention, and monthly for up to 6 months after completion of the intervention.
3. *Feasibility*: protocol deviation (PD) or study protocol implementation problems (eg, logistical).
4. *Usability*: participants’ experience using the system (perceived benefits, frustrations and recommendations) will be explored via exit interviews for all TeleRehaB DSS group completers. These participants will also complete the SUS^68^ and UEQ^69^ to explore system usability and user experience, respectively.

#### Secondary outcome measures

Various secondary outcome measures will be used to assess clinical outcomes, programme effectiveness, system performance, AI model predictive power, e-health literacy and user experience. Please see table 2 for the detailed schedule of assessments.

**Table 2:** Schedule of assessments for all participants enrolled in the TeleRehaB DSS study

		Screening	Baseline	Week 0	Weekly	Weeks 3 and 6	Post-intervention (week 10)	Monthly
Recruitment procedures	PIS informed consent	X	X					
Data collection and outcome measures	Demographics		X					
	HADS	X					X	
	DHI	X					X	
	FGA	X					X	
	MoCA	X					X	
	EQ-5D-5L		X				X	
	RAPA		X				X	
	ABC		X				X	
	Mini-BESTest		X				X	
	SVV		X				X	
	WEMWBS		X				X	
	FSS		X				X	
	SVQ		X				X	
	Exit interviews						X	
User experience measures	SUS						X	
	UEQ						X	
eHealth literacy measures	eHEALS		X					
	STAM-14		X					
	MDPQ-s		X					
Home-based interactions	Equipment installation and practice session (*IG only*)			X				
	Equipment de-installation (*IG only*)						X	
	Telephone call				X			
	Remote programme review					X		
	Perform HEP				X			
	Submit falls diary				X			X
	Submit exercise log				X			

ABC, Activities Specific Balance Confidence Scale; DHI, Dizziness Handicap Inventory; eHEALS, eHealth Literacy Assessment; EQ-5D-5L, EuroQol five-dimensional descriptive system; FGA, Functional Gait Assessment; FSS, Fatigue Severity Scale; HADS, Hospital Anxiety and Depression Scale; HEP, home exercise programme; IG, intervention group; MDPQ-s, Mobile Device Proficiency Questionnaire - short; Mini-BESTest, Mini-Balance Evaluation Systems Test; MoCA, Montreal Cognitive Assessment; PIS, Participant Information Sheet; RAPA, Rapid Assessment of Physical Activity; STAM, Senior Technology Acceptance and Adoption Model-14; SUS, System Usability Scale; SVQ, Situational Vertigo Questionnaire; SVV, Subjective Visual Verticality; TeleRehaB DSS, TeleRehabilitation Decision Support System; UEQ, User Experience Questionnaire; WEMWBS, The Warwick-Edinburgh Mental Wellbeing Scale.

Cost-effectiveness by means of the incremental cost-effectiveness ratio (ICER; the difference in mean costs divided by the difference in mean QALYs at the time of recruitment to the study and 1 year before the study and 1 year after the end of the intervention).

Objective measures include the Mini-Balance Evaluation Systems Test^70^ (mini-BESTest) to assess complex gait and dynamic balance tasks, the Montreal Cognitive Assessment (MoCA)^71^ as a measure of cognitive function, and the Subjective Visual Verticality (SVV)^72 73^ to evaluate a person’s perception of verticality. The mini-BESTest is a 14-item test that assesses static and dynamic balance, with a MDC of 3.5 and MCID of 4 points for balance/vestibular disorders^74^ and 5.52 points’ MDC in neurological conditions.^75^ The MoCA assess visuospatial/executive function, naming, attention, language, abstraction, memory and orientation to time and place, with a MDC of 4 points among older adults.^76^ SVV evaluates a person’s perception of verticality using a ‘rod and disk’ programme delivered on the computer, where the subject is asked to adjust a visual line to what they perceive as vertical. It is commonly used to diagnose and assess vestibular function.^72 73^

Subjective questionnaires include the Dizziness Handicap Inventory, a 25-item questionnaire which measures dizziness disability, with a MDC of 17.18 points and MCID of 18 points for balance/vestibular disorders;^77^ Activities-Specific Balance Confidence Scale,^78^ a 16-item questionnaire assessing balance confidence in varying situations, with a MDC of 14.89 in older adults,^79^ MDC of 19.79 points and MCID from 30.93 to 49.56 in stroke^80^ and MCID of 18 points in balance/vestibular disorders;^81^ Rapid Assessment of Physical Activity,^82^ a 9-item questionnaire to broadly assess physical activity levels; the Hospital Anxiety and Depression Scale,^83^ a 14-item scale which assesses anxiety and depression symptoms with an MDC of 4.66 and MCID of 1.17–2.13 for the anxiety subscale and MDC of 4.43 and MCID 1.48–2.54 for the depression subscale among patients with chronic pain;^84^ Fatigue Severity Scale,^85^ a 9-item instrument designed to measure fatigue, with a MDC of 1.9 points in multiple sclerosis,^86^ which will only be administered to the long covid cohort; the Warwick-Edinburgh Mental Wellbeing Scale,^87^ a 14-item scale assessing mental well-being; and the Situational Vertigo Questionnaire,^88^ a 20-item scale for assessing the impact of disorienting environments.

E-health literacy will be measured at baseline only, using the eHealth Literacy Assessment^89^ to evaluate patients’ skills in finding, evaluating and applying electronic health information; the Senior Technology Acceptance and Adoption Model-14^90^ to measure factors influencing technology acceptance among older adults; and the Mobile Device Proficiency Questionnaire - short,^91^ a highly reliable and valid measure of mobile device proficiency.

### Data management and analysis

#### Statistical analysis

Anonymised data will be entered into a study database. Data integrity will be assessed by taking a random 10% sample of entries following completion of data collection. The rectified database will be saved under a new filename (eg, study database_rectified_date) and all changes made to the database will be logged. All essential trial documentation will be saved on the dashboard except for the consent form which will be saved at each clinical site in paper form. The sponsor will ensure that study documentation is retained in accordance with their local approvals after the conclusion of the trial.

As this is a feasibility study, there is no formal plan for statistical analysis. However, for a future trial, an analysis plan has been developed which will be run in this study to determine its appropriateness. Descriptive statistics will be used to summarise feasibility outcomes of adherence, compliance and dropout rates. Continuous secondary outcome measures will be reported as means with 95% CIs.

Normality of outcome variables will be assessed by visual inspection and appropriate statistical tests. Where assumptions of normality are met, intention-to-treat analyses will be conducted using repeated-measures general linear model analysis of variance to explore between-group and within-group trends, adjusting for clinical site and cohort. Where normality assumptions are not met, non-parametric alternatives will be employed, including Wilcoxon signed-rank tests for within-group comparisons, Mann-Whitney U test for between-group comparisons and Friedman tests for repeated measures where appropriate.

#### Quality-adjusted life years

Health-related QOL will be assessed using the EQ-5D-5L at baseline and post-intervention, with utility values derived from country-specific tariffs. The area under the curve method will estimate QALYs over the intervention period, reflecting the intervention’s impact on participants’ quality and quantity of life.

#### Cost-effectiveness analysis (incremental cost-effectiveness ratio)

Total costs for the intervention and control groups will be calculated by summing the following: (1) intervention-specific costs (ie, equipment, technology and software costs); (2) standard care costs (printing and distribution costs of OTAGO HEP and Meniere’s Dizziness booklet, estimated therapist time and healthcare facility usage for standard care participants); and (3) indirect costs of data utilisation of healthcare records 1 year post-intervention to capture primary care services and hospital usage. The *ICER* will be calculated as follows:


$$ICER=\frac{\Delta Cost}{\Delta QALYs}$$

where ΔCost is the difference in average costs between the intervention and control groups, and QALYs is the difference in QALYs gained. To assess the robustness of the ICER, a one-way sensitivity analysis will vary key inputs, such as utility values, cost assumptions and healthcare utilisation rates, to evaluate their impact on the ICER. This will help identify variables with the greatest impact on cost-effectiveness.

#### Statistical approach for quality-adjusted life year and cost-effectiveness analysis

A mixed-effects regression model will compare QALYs and costs between groups, adjusting for confounders like age, baseline health and site. Cost-effectiveness will be evaluated against willingness-to-pay thresholds (eg, £20 000–£30 000 per QALY in the UK) to determine if TeleRehaB DSS is a cost-effective alternative to standard care.

#### Usability and acceptability

System effectiveness and user satisfaction with TeleRehaB DSS will be assessed by comparing predicted versus observed outcomes, with SUS and UEQ scores providing usability and user experience feedback.

Participants in the TeleRehaB DSS intervention group will be invited to provide qualitative feedback through exit interviews. Questions will cover user satisfaction, perceived effectiveness of TeleRehaB DSS and any challenges faced. Recorded and transcribed interviews will be analysed to identify recurring themes related to the ease of use, perceived benefits and any barriers to engaging with the system. Feedback from exit interviews will also be reviewed for insights into potential system improvements, such as interface usability, feature enhancements and suggestions for additional support or training.

## Discussion

This manuscript outlines the TeleRehaB DSS pilot feasibility protocol, a global, multisite RCT spanning clinical sites in the UK, Greece, Germany, Madeira and Thailand. Following a remote 9-week MBR programme delivered to adults at risk of falls due to stroke, MCI, vestibular dysfunction or long covid, the study aims to (1) determine the system’s safety, acceptability and feasibility; (2) assess how QOL, balance function and confidence, cognitive function and well-being compare to standard of care; and (3) provide preliminary data for a definitive RCT.

Preliminary findings from the HOLOBalance project, TeleRehaB DSS’ predecessor, support that a remotely delivered MBR is feasible to implement and acceptable to older adults at risk of falls, with balance and gait outcomes (FGA and Mini-BESTest) exceeding those from the OTAGO programme.^47^ Therefore, we hypothesise that the TeleRehaB DSS, a refined iteration of HOLOBalance, will be feasible to implement and acceptable to adults with stroke, MCI, vestibular dysfunction and long covid, although we do expect to receive disease-specific feedback from these patient cohorts, informing disease-specific refinements required to address each cohort’s needs. For example, patients who had a stroke commonly present with hemiplegia on one side of the upper and/or lower limbs, warranting exercise modifications to address these limitations. We also expect that MCI cohorts may require additional reminders and motivational prompts due to cognitive challenges such as memory. We expect the system to be safe, with minimal adverse events, as demonstrated in HOLOBalance (n=3 adverse events).

Compared with standard of care (OTAGO or Balance Retraining Booklet), we expect to see improved QOL, balance function, balance confidence and well-being in the TeleRehaB DSS intervention arms. HOLOBalance findings support this hypothesis, with FGA and Mini-BESTest findings meeting or approaching the MCID in the intervention group.^47^ This first iteration of the current TeleRehaB DSS demonstrates that provision of supervised remote home treatment for MBR provides notable benefits at reducing falls risks.^47^ Therefore, we expect that a more mature version will result in equal to if not better results following an extended treatment time of 9 weeks with additional exercises, improved instructions and ICT platforms, including hardware and software updates.

Final analysis of the target 460 participants across five multinational clinical sites, including a cost-benefit analysis, will hopefully provide preliminary data to support a fully powered definitive RCT of the TeleRehaB DSS. If so, we hope to determine its efficacy as a falls prevention and rehabilitation clinical tool that can be successfully implemented into local healthcare systems.

### Monitoring

#### Study oversight

Trial steering committees and trial management groups will be formed to oversee the conduct of the research according to predetermined terms of reference.

#### Harms

A safety reporting protocol exists for reporting of research incidents, including adverse events, device deficiencies and PDs. All clinical sites will undergo safety reporting training.

### Patient and public involvement

As part of the larger TeleRehaB DSS project, the research consortium has performed extensive patient and public involvement across the clinical sites (University College London, University Medical Centre Freiberg Neurocenter, National Kapodistrian University of Athens, King Chulalongkorn Memorial Hospital) with over 30 older adult patients who had a stroke and carers, patients with MCI and patients with vestibular dysfunction; over 300 public members and greater than 50 clinicians. A mixture of hybrid (face-to-face and remote) interviews, focus groups and workshops discussing and gathering qualitative and quantitative feedback pertaining to the study design, dashboard, mobile app, exergames and the supporting technologies was collected. This feedback was collected, analysed and implemented into the design of the TeleRehaB DSS as a patient-centric and user-friendly platform.

## Ethics and dissemination

This study involves human participants and was approved by ethics committee(s) or institutional board(s) at all clinical sites: the UK (UCL Research Ethics Committee, ID: 17413/002), Germany (Ethik-Kommission Freiburg, ID: 23-1539_1-S1), Greece (The Ethics Committee, ‘Hippokrateion’ Hospital, ID: 21855) and Thailand (The Institutional Review Board of the Faculty of Medicine, Chulalongkorn University, ID: IRB 054/66) and Madeira, Portugal (Serviço de Saúde da RAM, EPERAM, ID: S. 25004510).

Findings from this study will be submitted for peer-reviewed publications. All amendments to the study protocol which may impact the integrity of the study or the data are required to receive approval by the research ethics committee prior to their implementation.

Findings from this study will be provided as lay reports to participants, disseminated to community groups via community partners and submitted for peer-reviewed publications. Additionally, electronic data will be anonymised and uploaded to a data repository that supports restricted access. Electronic data will not be made publicly available and access to the dataset will only be provided by the data management board of the TeleRehaB DSS research consortium. Use and reuse of the pilot dataset will be subject to the licence under which the data objects were deposited.

## Supplementary material

10.1136/bmjopen-2025-108821 online supplemental file 1

10.1136/bmjopen-2025-108821 online supplemental file 2
