# Supplementary material for: Investigating the feasibility and acceptability of the TeleRehabilitation of balance clinical and economic Decision Support System (TeleRehaB DSS) in adults at risk of falls: study protocol for a multicentre clinical trial
Source: BMJ Open. 2026 Jun 25;16(6):e108821. doi: 10.1136/bmjopen-2025-108821 (PMC13311700; doi:10.1136/bmjopen-2025-108821)
Supplement: online supplemental file 1 [file bmjopen-16-6-s001.pdf]

Appendix 1 – Consent Form

CONSENT FORM FOR PARTICIPANTS IN RESEARCH STUDIES

Please complete this form after you have read the Information Sheet and/or listened to an explanation about the research.

Title of Study: TELEREHABILITATION OF BALANCE CLINICAL AND ECONOMIC DECISION SUPPORT SYSTEM (Randomised Controlled Trial)

Department: Centre for Vestibular and Behavioural Neurosciences, Department of Clinical and Motor Neurosciences, 5th Floor, 33 Queen Square, London WC1N 3BG

Name and Contact Details of the Researcher(s):

Brooke Nairn, email: b.nairn@ucl.ac.uk

Dr Nehzat Koohi, email: *************

Name and Contact Details of the Principal Researcher:

Professor Doris-Eva Bamiou, email: d.bamiou@ucl.ac.uk

Name and Contact Details of the UCL Data Protection Officer: Alexandra Potts data-protection@ucl.ac.uk

This study has been approved by the UCL Research Ethics Committee: Project ID number: 17413/002

Thank you for considering taking part in this research. The person organising the research must explain the project to you before you agree to take part. If you have any questions arising from the Information Sheet or explanation already given to you, please ask the researcher before you decide whether to join in. You will be given a copy of this Consent Form to keep and refer to at any time.

I confirm that I understand that by ticking/initialling each box below I am consenting to this element of the study. I understand that it will be assumed that unticked/initialled boxes means that I DO NOT consent to that part of the study. I understand that by not giving consent for any one element that I may be deemed ineligible for the study.

	Initial Box
I voluntarily agree to take part in this study.	
I confirm that I have read and understood the Information Sheet (Version 3.0, dated 31/10/2024) for the above study. I have had an opportunity to consider the information and what will be expected of me. I have also had the opportunity to ask questions which have been answered to my satisfaction.	
I understand that I have the right to can withdraw from the study at any stage however pseudonymised data collected up to that time may be used for further research analysis (according to article 28(3) of the CTR).	
I understand that I may be withdrawn from the study, as per the terms outlined in the participant information sheet.	
I consent to participate in the study. I understand that my personal information will be used for the purposes explained to me. I understand that according to data protection legislation (GDPR), use of my data is legally allowed, given:<br>▪ consent must be freely given and only open to one interpretation<br>▪ it is of the interest to the public for health purposes.<br>▪ The processing of health data is for scientific purposes	

I understand that all personal information will remain confidential and that my data collected for this study will be stored securely. I understand that all efforts will be made to ensure I cannot be identified in any publications.	
I understand that my information may be subject to review by responsible individuals from University College London for monitoring and audit purposes.	
I understand the potential risks of participating in the study. I understand that the support that will be available to me, should I become distressed during the research, and how to seek help.	
I understand the direct and indirect benefits of participating in this study.	
I understand that the data will not be made available to any commercial organisations outside of the research project, and is solely the responsibility of the researcher(s) undertaking this study.	
I understand that I will not benefit financially from this study or from any possible outcome it may result in in the future.	
I understand that I will be compensated for the travel expenses incurred getting to and from the research centre.	
I understand that the information I have submitted will be published as a report	
I wish to receive a copy of the published report	
I do not wish to received a copy of the published report	
I hereby confirm that:<br>(a) I understand the exclusion criteria as detailed in the Information Sheet and explained to me by the researcher;<br>AND<br>(b) I do not fall under the exclusion criteria.	
I agree to my GP being informed of my participation in the study.	
I agree to my GP being informed if my questionnaire scores indicate increased anxiety and/or depression scores.	
I agree that my GP may be contacted if any unexpected results are found in relation to my health.	
I have informed the researcher of any other research in which I am currently involved or have been involved in during the past 12 months.	
I am aware of who I should contact if I wish to lodge a complaint.	
I agree for the program's information technology, software applications and hardware devices to be set up in my home.	
I agree to the remote access program to be set- up in my home	
I understand that the data I provide will be archived in pseudonymised form at University College London for the purpose of additional data analyses for 20 years, beyond the end of the study. I understand that other authenticated researchers will have access to my pseudonymised data.	
I understand, that GARMIN will be used as a sub-processor in order for my data to be collected, for the clinical study, and all efforts will be made to ensure my privacy, and security of my data.	
I understand that my personal data will not be transferred overseas. I understand that only pseudonymised data collected in this study will be transferred to the research consortium members within the EEA using an encrypted dedicated cloud storage.	

Pseudonymised:** *data is personal data that has been altered in such a way that it* ***can no longer be directly attributed to an individual without the use of additional information *(such as a key or code). The data is still linked to an individual, but the identifying information is replaced or masked (e.g., using a reference number or code).*

If you would like your contact details to be retained so that you can be contacted in the future by UCL researchers who would like to invite you to participate in follow up studies to this project, or in future studies of a similar nature, please tick the appropriate box below.

	Yes, I would be happy to be contacted in this way	
	No, I would not like to be contacted	

Name of participant ________________ Date ________________ Signature ________________

Researcher Statement (when discussions have taken place)

I ………………………………………………………... confirm that I have carefully explained the purpose of the study to the participant and outlined any reasonably foreseeable risks or benefits (where applicable).

Researcher ________________ Date ________________ Signature ________________
