# Supplementary material for: Investigating the feasibility and acceptability of the TeleRehabilitation of balance clinical and economic Decision Support System (TeleRehaB DSS) in adults at risk of falls: study protocol for a multicentre clinical trial
Source: BMJ Open. 2026 Jun 25;16(6):e108821. doi: 10.1136/bmjopen-2025-108821 (PMC13311700; doi:10.1136/bmjopen-2025-108821)
Supplement: online supplemental file 2 [file bmjopen-16-6-s002.pdf]

Supplemental Material: TeleRehab DSS Protocol

Table S1. TeleRehab DSS intervention balance exercise and their progressions

Exercise Name	*Exercise Description*	*Progression (P)*
Previous HOLOBalance Exercises		
Sitting 1 – Yaw head turns	Head turns side to side (yaw rotation of 30°) while visually fixating on a static target placed at eye level in front of the participant	P1: Increase speed of movements<br>P2: Visual target moves in opposite direction to head movement<br>P3: Increase speed of P2
Sitting 2 – Pitch head tilts	Head turns up and down (pitch rotation of 30°) while visually fixating on a static target placed at eye level in front of the participant	P1: Increase speed of movements<br>P2: Look up to ceiling and down to floor without fixation<br>P3: Increase speed of P2
Sitting 3 – Bend over	Bend as if to pick up an object off the floor from sitting position and return to upright position	P1: Perform with eyes closed<br>P2: Reach to the side to pick up objects<br>P3: Perform P2 with eyes closed
Standing 1 – Maintain balance	Stand with FHWA, looking straight ahead with eyes open	P1: FCT<br>P2: FHWA with eyes closed<br>P3: FCT, eyes closed
Standing 2 – Maintain balance on foam	Stand on foam cushion with FHWA and looking straight ahead with eyes open	P1: FCT<br>P2: FHWA with eyes closed<br>P3: FCT, eyes closed
Standing 3 – Bend Over	Stand with FHWA and bend over to pick up an object from the floor	P1: FCT
Standing 4 – Overhead Reach	Stand with FHWA and reach over head to grab an object out of the cupboard	P1: Add a heel lift
Standing 5 – Turn	Step turn through 180° to face the opposite direction	P1: Increase speed of movement
Walking 1 - Walk to horizon	Walk across the room in a straight path while looking at the horizon	P1: Increase speed
Walking 2 – Walk & Yaw	Walk forwards across the room looking between two targets placed at eye level, 1.5-meters apart on the horizontal plane	P1: Increase walking and head turn speed<br>P2: Increase head turn range and scan the room<br>P3: Increase head turn range to P2, as if crossing the street.

Walking 3 – Walk & Pitch	Walk across the room and nod your head to look up to the ceiling and down to the ground	P1: Increase walking and head turn speed<br>P2: Integrate diagonal 'V' head<br>Movements<br>P3: Increase speed of P2
New Exercises		
Sitting 4 – Seated trunk rotation	Turn around to look behind you	P1: increase speed<br>P2: increase range
Sitting 5 – Toe raises	Lift your toes off the ground	P1: perform unassisted
Sitting 6 – Heel raises	Lift your heels off the ground	P1: perform in standing
Sitting 7 – Seated marching on the spot	Alternate lifting one knee at a time as if marching	P1: add knee hold<br>P2: add arm swing
Sitting 8 – Sit to stand	From a seated position, stand up, and sit back down	P1: 1-arm assisted<br>P2: unassisted
Standing 6 – Lateral weight shifts	Shift side to side from the trunk to the ankles	P1: Bring feet closer together
Standing 7 – Limits of stability	Lean forwards and backwards from the trunk to the ankles	P1: Increase speed
Standing 8 – Forward reach	Lean forwards and backwards from the trunk to the ankles	P1: Increase speed
Walking 4 – Side Stepping	Step side to step	P1: Increase speed
Stretching 1 - Hip external rotator stretch	In sitting, cross your foot over your knee and lean forward	P1: lean further into stretch
Stretching 2 - Lateral trunk flexion	In sitting, lean to the side and hold	P1: lean further into stretch
Stretching 3 - Calf stretch	In standing, place your toes on the edge of step and lean forward	P1: lean further into stretch
Sitting - Optokinetic Video	In sitting, look through the screen	P1: yaw head turns
Standing – Optokinetic Video	In standing, look through the screen	P1: yaw head turns
Exergame - Sit - Yaw	Bird follow game - heard turning left and right	

Exergame - Sit - Pitch	Bird follow game - heard turning up and down
Exergame - Stand - Maintain Balance	Seal juggle game in standing
Exergame - Stand - Bend	Dog command lay down game
Exergame - Stand - Reach	Dog command jumping game
Exergame - Walk - Horizon	Bird to flower walking game
Exergame - Walk - Yaw	Bird to flower walking head turn left and right game
Exergame - Walk - Pitch	Bird to flower walking head tilt up and down game
Animal Feeding	Reaction Time<br>Visuospatial<br>Rapid Visual Information Processing
Bridge Crossing	Visuospatial<br>Attention
Catching Food	Reaction Time<br>Rapid Visual Information Processing
Memory Game	Memory
Preparing Animal Food	Reaction Time<br>Rapid Visual Information Processing
Remember Previous Game	Memory<br>Rapid Visual Information Processing

*Feet close together (FCT); Feet hip width apart (FHWA); Feet shoulder width apart (FSWA); Eyes closed (EC); Eyes open (EO)

Table S2. Weekly falls diary during intervention

Week 1: *17/06/2024- 23/06/2024*								
Weekly Falls Diary		**Mon**	**Tues**	**Wed**	**Thurs**	**Fri**	**Sat**	**Sun**
	No Fall							
	Near fall							

**If you fell, what injury did you have, if any?**	No injury							
	Bruise or cut							
	Muscle or ligament							
	Broken bone							

Appendix 1 – Consent Form

CONSENT FORM FOR PARTICIPANTS IN RESEARCH STUDIES

Please complete this form after you have read the Information Sheet and/or listened to an explanation about the research.

Title of Study: TELEREHABILITATION OF BALANCE CLINICAL AND ECONOMIC DECISION SUPPORT SYSTEM (Randomised Controlled Trial)

Department: Centre for Vestibular and Behavioural Neurosciences, Department of Clinical and Motor Neurosciences, 5th Floor, 33 Queen Square, London WC1N 3BG

Name and Contact Details of the Researcher(s):

Brooke Nairn, email: b.nairn@ucl.ac.uk

Dr Nehzat Koohi, email: ********************

Name and Contact Details of the Principal Researcher:

Professor Doris-Eva Bamiou, email: d.bamiou@ucl.ac.uk

Name and Contact Details of the UCL Data Protection Officer: Alexandra Potts data-protection@ucl.ac.uk

This study has been approved by the UCL Research Ethics Committee: Project ID number: 17413/002

Thank you for considering taking part in this research. The person organising the research must explain the project to you before you agree to take part. If you have any questions arising from the Information Sheet or explanation already given to you, please ask the researcher before you decide whether to join in. You will be given a copy of this Consent Form to keep and refer to at any time.

I confirm that I understand that by ticking/initialling each box below I am consenting to this element of the study. I understand that it will be assumed that unticked/initialled boxes means that I DO NOT consent to that part of the study. I understand that by not giving consent for any one element that I may be deemed ineligible for the study.

	Initial Box
I voluntarily agree to take part in this study.	
I confirm that I have read and understood the Information Sheet (Version 3.0, dated 31/10/2024) for the above study. I have had an opportunity to consider the information and what will be expected of me. I have also had the opportunity to ask questions which have been answered to my satisfaction.	
I understand that I have the right to can withdraw from the study at any stage however pseudonymised data collected up to that time may be used for further research analysis (according to article 28(3) of the CTR).	
I understand that I may be withdrawn from the study, as per the terms outlined in the participant information sheet.	
I consent to participate in the study. I understand that my personal information will be used for the purposes explained to me. I understand that according to data protection legislation (GDPR), use of my data is legally allowed, given:<br>▪ consent must be freely given and only open to one interpretation<br>▪ it is of the interest to the public for health purposes.<br>▪ The processing of health data is for scientific purposes	
I understand that all personal information will remain confidential and that my data collected for this study will be stored securely. I understand that all efforts will be made to ensure I cannot be identified in any publications.	
I understand that my information may be subject to review by responsible individuals from University College London for monitoring and audit purposes.	

I understand the potential risks of participating in the study. I understand that the support that will be available to me, should I become distressed during the research, and how to seek help.	
I understand the direct and indirect benefits of participating in this study.	
I understand that the data will not be made available to any commercial organisations outside of the research project, and is solely the responsibility of the researcher(s) undertaking this study.	
I understand that I will not benefit financially from this study or from any possible outcome it may result in in the future.	
I understand that I will be compensated for the travel expenses incurred getting to and from the research centre.	
I understand that the information I have submitted will be published as a report	
I wish to receive a copy of the published report	
I do not wish to received a copy of the published report	
I hereby confirm that:<br>(a) I understand the exclusion criteria as detailed in the Information Sheet and explained to me by the researcher;<br>AND<br>(b) I do not fall under the exclusion criteria.	
I agree to my GP being informed of my participation in the study.	
I agree to my GP being informed if my questionnaire scores indicate increased anxiety and/or depression scores.	
I agree that my GP may be contacted if any unexpected results are found in relation to my health.	
I have informed the researcher of any other research in which I am currently involved or have been involved in during the past 12 months.	
I am aware of who I should contact if I wish to lodge a complaint.	
I agree for the program's information technology, software applications and hardware devices to be set up in my home.	
I agree to the remote access program to be set- up in my home	
I understand that the data I provide will be archived in pseudonymised form at University College London for the purpose of additional data analyses for 20 years, beyond the end of the study. I understand that other authenticated researchers will have access to my pseudonymised data.	
I understand, that GARMIN will be used as a sub-processor in order for my data to be collected, for the clinical study, and all efforts will be made to ensure my privacy, and security of my data.	
I understand that my personal data will not be transferred overseas. I understand that only pseudonymised data collected in this study will be transferred to the research consortium members within the EEA using an encrypted dedicated cloud storage.	

Pseudonymised:** *data is personal data that has been altered in such a way that it* ***can no longer be directly attributed to an individual without the use of additional information *(such as a key or code). The data is still linked to an individual, but the identifying information is replaced or masked (e.g., using a reference number or code).*

If you would like your contact details to be retained so that you can be contacted in the future by UCL researchers who would like to invite you to participate in follow up studies to this project, or in future studies of a similar nature, please tick the appropriate box below.

	Yes, I would be happy to be contacted in this way	

	No, I would not like to be contacted	

Name of participant ______________________ Date ______________ Signature ________________

Researcher Statement (when discussions have taken place)

I ……………………………………………………... confirm that I have carefully explained the purpose of the study to the participant and outlined any reasonably foreseeable risks or benefits (where applicable).

Researcher ________________ Date ______________ Signature ________________
