# Supplementary material for: Investigating the feasibility and acceptability of the TeleRehabilitation of balance clinical and economic Decision Support System (TeleRehaB DSS) in adults at risk of falls: study protocol for a multicentre clinical trial
Source: BMJ Open. 2026 Jun 25;16(6):e108821. doi: 10.1136/bmjopen-2025-108821 (PMC13311700; doi:10.1136/bmjopen-2025-108821)
Supplement: Supplementary file 3 [file bmjopen-2025-108821.reviewer_comments.pdf]

PEER REVIEW HISTORY

BMJ Open publishes all reviews undertaken for accepted manuscripts. Reviewers are asked to complete a checklist review form and are provided with free text boxes to elaborate on their assessment. These free text comments are reproduced below.

ARTICLE DETAILS

Title (Provisional)

Investigating the feasibility and acceptability of the TeleRehabilitation of balance clinical and economic Decision Support System (TeleRehab DSS) in adults at risk of falls: study protocol for a multicenter clinical trial

Authors

Nairn, Brooke; Walz, Isabelle; Nikitas, Christos; Kaski, Diego; Utoomprurkporn, Nattawan; Maurer, Christoph; Kikidis, Dimitris; Tsakanikas, Vassilios; Fotiadis, Dimitrios; Pavlou, Marousa; Bamiou, Doris-Eva

---

VERSION 1 - REVIEW

**Reviewer**	**1**
**Name**	**Jiménez-Sánchez, Carolina**
**Affiliation**	**University of San Jorge Faculty of Health Sciences, Department of Physiotherapy**
**Date**	**23-Dec-2025**
**COI**	**None**

General comments:

Interesting paper on Investigating the feasibility and acceptability of the TeleRehabilitation of balance clinical and economic Decision Support System (TeleRehab DSS) in adults at risk of falls: study protocol for a multicenter clinical trial. My major concerns deal with content. It is necessary to review important aspects along the manuscript in order to be taken into account for publication.

- **Abstract**
  - Authors should modify Abstract section if necessary, after manuscript revision.

- **Introduction**
  - Lines 19-21, page 7. I think that introducing telerehabilitation here is somewhat forced. I think it would be more appropriate in the following paragraph, where the justification for using this rehabilitation format can be addressed.
  - Line 28, page 7. The Telerehabilitation. The letter t in telerehabilitation should be lowercase.

- Given the relevance of telerehabilitation, I believe that an adequate definition and further explanation of the types of telerehabilitation are lacking.
- E-health technology solutions, page 7. The introduction should discuss previous experiences of telerehabilitation in the treatment of balance; this section should not primarily introduce the concept of the study. It should justify the study and refrain from drawing conclusions about its effectiveness.
- Aims, page 7. The objectives should be the project objectives in terms of the feasibility and effectiveness of the intervention. The objectives set for the TeleRehab DSS intervention should not be included because this protocol is a randomised clinical trial against another intervention that could also be effective. The first part of this subsection should be modified by removing information that does not directly refer to the study objectives.

- **Methods**
  - Trial design
    - Line 30, page 8. OTAGO HEP or Meniere's Dizziness Booklet?
    - There are three armss: low technology, high technology, and control. This must also be reflected in the study design.
  - Sample size
    - How was the number of documented participants determined?
  - Intervention
    - The TeleRehab DSS IG? If the technology group is the intervention group, IG, use that acronym, do not write out the full name.
    - Each technology group should be given a specific name, and once the acronyms have been defined, they should be used throughout the manuscript.
    - Line 39, page 11. semi-structured interview… For what purpose? Is it worthwhile to publish these qualitative results? This must be made clear in the protocol throughout the manuscript.
    - Is there a difference in prescription patterns between the intervention groups and the control group? This must be taken into account because it may influence a clear comparison.
  - Outcome measures
    - You must specify the variables that will only be used with the IG group.
    - Each variable should be explained in more detail with its interpretation and, in the case of secondary variables, if possible, it would be very interesting to indicate the minimum detectable change.
  - Statistical analysis
    - The only thing I think is missing from the statistical analysis is an indication that the normality of the variables will be assessed and non-parametric tests will be considered if necessary.

- **Discussion section.**
  - I am surprised that there is no Discussion section. Even though there are no results, nowadays protocols usually include a discussion of possible future findings. In fact, the vast majority of protocol articles published in BMJ Open include a Discussion section. I believe this is essential for a manuscript to be considered publishable.

VERSION 1 - AUTHOR RESPONSE

Reviewer: 1
Dr. Carolina Jiménez-Sánchez, University of San Jorge Faculty of Health Sciences
Comments to the Author:
General comments:
Interesting paper on Investigating the feasibility and acceptability of the TeleRehabilitation of balance clinical and economic Decision Support System (TeleRehab DSS) in adults at risk of falls: study protocol for a multicenter clinical trial. My major concerns deal with content. It is necessary to review important aspects along the manuscript in order to be taken into account for publication.

• Abstract
o Authors should modify Abstract section if necessary, after manuscript revision.
RESPONSE: Thank you, the abstract has been modified according to journal guidelines.

• Introduction
o Lines 19-21, page 7. I think that introducing telerehabilitation here is somewhat forced. I think it would be more appropriate in the following paragraph, where the justification for using this rehabilitation format can be addressed.

RESPONSE: Thank you, this has been moved to the following paragraph, under heading digital health technologies.

o Line 28, page 7. The Telerehabilitation. The letter t in telerehabilitation should be lowercase.
RESPONSE: Thank you, this has been corrected.
o Given the relevance of telerehabilitation, I believe that an adequate definition and further explanation of the types of telerehabilitation are lacking.
RESPONSE: Thank you, we have endeavoured to better define telerehabilitation and other digital health technologies, under the sub-section: digital health technologies (page 5, lines 2-19)

o E-health technology solutions, page 7. The introduction should discuss previous experiences of telerehabilitation in the treatment of balance; this section should not primarily introduce the concept of the study. It should justify the study and refrain from drawing conclusions about its effectiveness.
RESPONSE: Thank you for this feedback. We have included additional information, referencing other telerehabilitation treatments for balance, along with its benefits to both clinicians and patients, supporting it's use. (page 5, lines 5 - 13)

o Aims, page 7. The objectives should be the project objectives in terms of the feasibility and effectiveness of the intervention. The objectives set for the TeleRehab DSS intervention should not be included because this protocol is a randomised clinical trial against another

intervention that could also be effective. The first part of this subsection should be modified by removing information that does not directly refer to the study objectives.
RESPONSE: Thank you for catching this, which we agree with. We have moved the more descriptive details to the section above, where we introduce the concept of TeleRehab, leaving just the objectives of the study under the 'Aims' section.

• Methods
o Trial design
 Line 30, page 8. OTAGO HEP or Meniere's Dizziness Booklet?
RESPONSE: Yes there are indeed two CG arms. We have defined the two groups under the control groups section, and revised the wording to help with clarity (page 9, lines 12-14): CG participants will receive a standard-of-care balance rehabilitation HEP; Stroke and MCI participants will complete the OTAGO HEP 54,65, while vestibular and long covid-19 participants will complete the Balance Retraining Booklet 66,67 (Appendix 2 - Supplemental Materials).
We have also included links to these booklets in the Supplemental Materials.

 There are three arms: low technology, high technology, and control. This must also be reflected in the study design.
RESEPONSE: Thank you, this has now been included the Trial Design section (page 5, lines 34-37): "All participants will be randomised into one of 3 study arms, where they will receive a HEP provided under the supervision of a clinician (physiotherapist or sports scientist): i) the high-technology TeleRehab DSS group (HT-IG), ii) the low-technology TeleRehab DSS group (LT-IG), or iii) the control group (CG) standard-of-care HEP. 5455"

o Sample size
 How was the number of documented participants determined?
RESPONSE:As a pilot feasibility RCT, sample size estimate was based on the literature's recommendation for pilot feasibility studies. Teresi et al. 2022 notes that sample size should be based on "practical considerations including participant flow, budgetary constraints, and the number of participants needed to reasonably evaluate feasibility goals." Whitehead et al. 2015 recommends that for quantitative studies, a sample of 30 per group (intervention and control) may be adequate to establish feasibility. We have amended the wording to reflect this (page 7, lines 22-24): "The target sample size across all sites is 460. This equates to 60-100 participants per clinical site, with 30-50 participants recruited to IG and CG study arms, per clinical site, in line with recommendations for pilot feasibility studies".59–61 61

oIntervention
 The TeleRehab DSS IG? If the technology group is the intervention group, IG, use that acronym, do not write out the full name.
RESPONSE: Yes, there is a high-tech intervention group (now labelled as HT-IG) and low-tech intervention group (now labelled as LT-IG). We have re-labelled with these acronyms throughout the revised manuscript for clarity, which we hope are now more appropriate.

 Each technology group should be given a specific name, and once the acronyms have been defined, they should be used throughout the manuscript.
RESPONSE: Thank you, as above, we have re-labelled with more appropriate acronyms for clarity: high-tech intervention group (HT-IG) and low-tech intervention group (LT-IG).

 Line 39, page 11. semi-structured interview… For what purpose? Is it worthwhile to

publish these qualitative results? This must be made clear in the protocol throughout the manuscript.
RESPONSE: To gather qualitative feedback on acceptability and usability. Thank you for highlighting this. We have added the below sentence to better explain the purpose of these semi-structured interviews (page 9, lines 8-10): “This qualitative feedback on acceptability and usability will help to inform future iterations of the platform, for a larger scale RCT and eventual adoption into local healthcare systems.”

 Is there a difference in prescription patterns between the intervention groups and the control group? This must be taken into account because it may influence a clear comparison.
RESPONSE: No, the prescription pattern is the same for IG and CG to maintain a clear comparison. We have clarified this in the revised text, thus (page 5, lines 37-40): “No matter the study arm allocated, all participants will received the same prescription pattern, receiving a weekly check-in call by a member of the research team, and remote program reviews at weeks 3 and 6 by the treating clinician, to discuss exercise modifications, symptoms, falls, and any technical problems.”.

o Outcome measures
 You must specify the variables that will only be used with the IG group.
RESPONSE: This is outlined in Table 2. Schedule of assessments

 Each variable should be explained in more detail with its interpretation and, in the case of secondary variables, if possible, it would be very interesting to indicate the minimum detectable change.
RESPONSE: We have updated all outcomes measures with a brief description while indicating either the MDC and/or MCID where data was available (pages 9-11). Despite extensive searches, minimum detectable changes are not, to our knowledge, available for SVV, WEMBS, RAPA or SVQ.

o Statistical analysis
 The only thing I think is missing from the statistical analysis is an indication that the normality of the variables will be assessed and non-parametric tests will be considered if necessary.
RESPONSE: Thank you for catching this. We did intend to include this and have updated the manuscript accordingly with this statement (page13 , lines 18-19): “Normality of the variables will be assessed and non-parametric tests will be considered if necessary.” We apologise for the oversight.

• Discussion section.
o I am surprised that there is no Discussion section. Even though there are no results, nowadays protocols usually include a discussion of possible future findings. In fact, the vast majority of protocol articles published in BMJ Open include a Discussion section. I believe this is essential for a manuscript to be considered publishable.
RESPONSE: Following this feedback, a discussion section of expected findings has been added (pages 13-14).

Reviewer: 1
If you have selected ‘Yes’ above, please provide details of any competing interests.: Not applicable

VERSION 2 - REVIEW

Reviewer 1

Name Jiménez-Sánchez, Carolina

Affiliation University of San Jorge Faculty of Health Sciences, Department of Physiotherapy

Date 25-Jan-2026

COI

• Methods

o Intervention

 Intervention Group (IG): TeleRehab DSS (high tech and low-tech intervention arms) Participants randomised into the TeleRehab DSS IG will undergo a second randomisation into either the 1) high-tech group, or 2) low-tech group.

If you have already established the names of the groups, those acronyms should be used.

o Statistical analysis

 Normality of the variables will be assessed and non-parametric tests will be considered if necessary. This sentence does not make sense at the end. It would be logical to place it before discussing the tests used, and the non-parametric analysis to be used should be included if it needs to be performed.

VERSION 2 - AUTHOR RESPONSE

Reviewer: 1
Dr. Carolina Jiménez-Sánchez, University of San Jorge Faculty of Health Sciences
Comments to the Author:
• Methods
o Intervention
 Intervention Group (IG): TeleRehab DSS (high tech and low-tech intervention arms) Participants randomised into the TeleRehab DSS IG will undergo a second randomisation into either the 1) high-tech group, or 2) low-tech group.
If you have already established the names of the groups, those acronyms should be used.
RESPONSE: Apologies for missing this. I have now changed this using the appropriately defined acronyms HT-IG and LT-IG.

Statistical analysis
 Normality of the variables will be assessed and non-parametric tests will be considered if necessary. This sentence does not make sense at the end. It would be logical to place it before discussing the tests used, and the non-parametric analysis to be used should be included if it needs to be performed.

RESPONSE: Thank you for this feedback. Please find the revised statistical analysis section below which includes mention of the normality testing earlier on, while defining the non-parametric test to be used (page 12, lines 21-27):
Normality of outcome variables will be assessed by visual inspection and appropriate statistical tests. Where assumptions of normality are met, intention-to-treat analyses will be conducted using repeated-measures general linear model analysis of variance to explore between-group and within-group trends, adjusting for clinical site and cohort. Where normality assumptions are not met, non-parametric alternatives will be employed, including Wilcoxon signed-rank tests for within-group comparisons, Mann-Whitney U test for between-group comparisons, and Friedman tests for repeated measures where appropriate.
